# Supplementary material for: Highly Sensitive Detection of Zika Virus Nonstructural Protein 1 in Serum Samples by a Two-Site Nanobody ELISA
Source: Biomolecules. 2020 Dec 9;10(12):1652. doi: 10.3390/biom10121652 (PMC7763430; doi:10.3390/biom10121652)
Supplement: Supplementary file 1 [file biomolecules-10-01652-s001.pdf]

biomolecules

MDPI

Supplementary Material

Highly sensitive detection of Zika virus non-structural protein 1 in serum samples by a two-site nanobody ELISA

Triana Delfin-Riela[1], Martín Rossotti[1], Romina Alvez-Rosado[1], Carmen Leizagoyen[2] and Gualberto González-Sapienza[1,*]

[1]Cátedra de Inmunología, DEPBIO, Facultad de Química, Instituto de Higiene, UDELAR, Montevideo, Uruguay. [2]Parque Lecoq, IMM, Montevideo Uruguay.

*Corresponding author. Av. A. Navarro 3051, 2nd floor. 11600 Montevideo, Uruguay, ggonzal@fq.edu.uy, (598) 24874334.

Keywords: Nanobody, Zika, NS1, Immunoassay/ELISA, Phage Display,

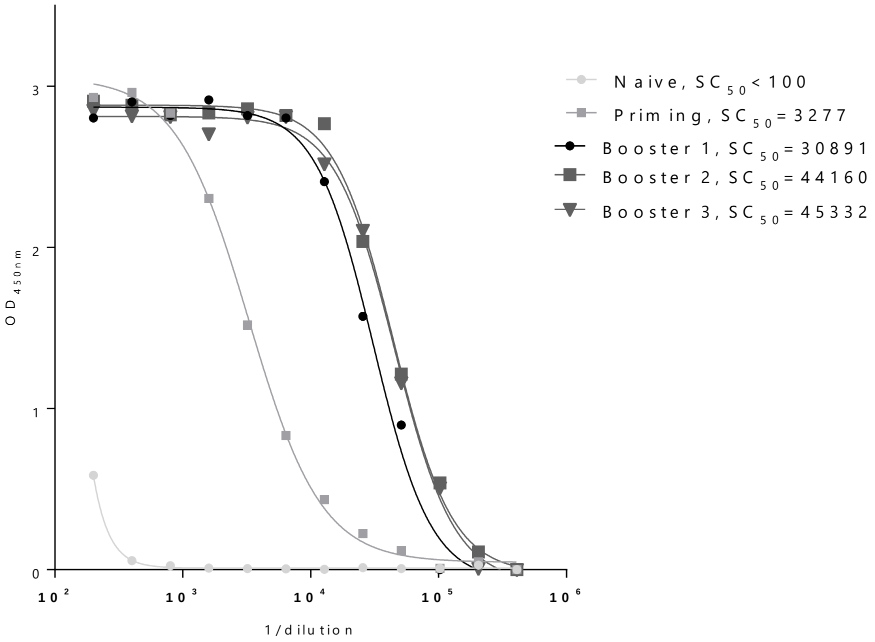

Figure S1. Serum titration against ZVNS1 along the immunization process. Serum samples were analyzed by ELISA on plates coated with ZVNS1. The titer, calculated as the concentration causing a 50% decrease in the maximum signal (SC_{50}) is shown in the insert and was used as an indicator of the progression of the llama immunity response.

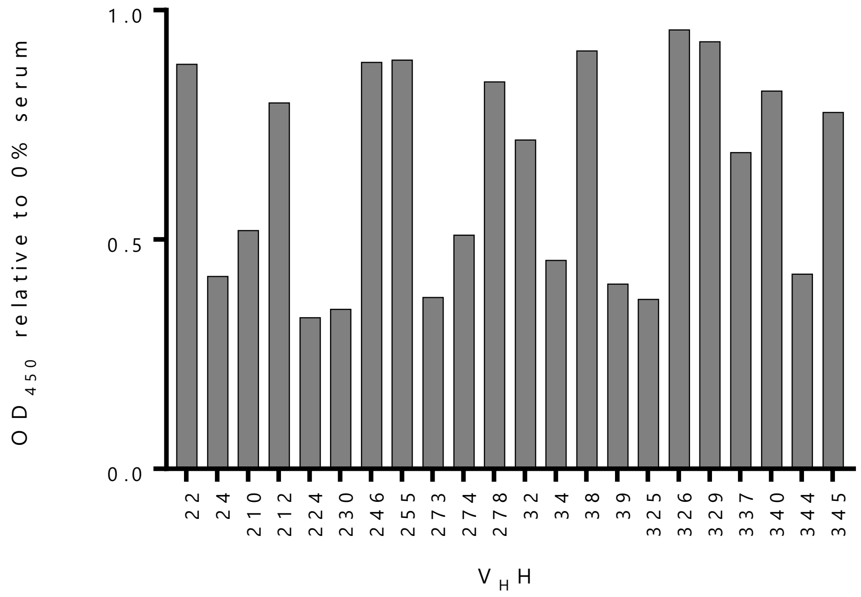

Figure S2- Performance of 22 Nb clones in the presence of human serum. The culture supernatant of each clone was assayed on wells coated with 100 ng/well of ZVNS1 in the presence or absence of 50% human serum. Bars represent the average of two measurements.

```
         ---------FR1-------------    CDR1      -----FR2---------    CDR2       -----------------FR3--------------------    CDR3            ----FR4----
1   22   EVQLVESGGGLVQTGGSLRLSCAAS GTIFSTKA MGWYRQAPGKRREFVAL IAPGGDI---- TYADSAEGRFTISRDSAKGTV--WLQMNDLKAEDTAVYYC NTVPRTQD-------- WGQGTQVTVSS
51 278   QVQLVQSGGGLVRPGGSLRLSCAAS GNIFSTKA MGWYRQAPGKRREFVAL IDPAGST---- TYADSVEGRFTISKDSAKGTV--WLQMNDLKAEDTAVYYC NTVPRVQD-------- WGQGTQVTVSS
50 274   EVQLVESGGGTVQTGGSLRLSCAAF GSIFSRKA VGWYRQAPGKQREFLAL IAPAGDT---- TYADSVEGRFTISRDSATNTV--WLQMNNLEPEDTAAYYC NTIPRVKD-------- WGQGTQVTVSS
17 337   EVQLVESGGGLVQTGGSLRLSCVTS GVIFSRSA VGWYRQAPGERREFVAL IDPAGTT-----TYADAVEGRFTISRDSAKNTV--WLQMNDLKAEDTSVYYC NTVPRIKD-------- WGQGTQVTVSS(2)
68 326   QVQLVQSGGGLVQAGGSLTLSCSDS GSIFRHNS MGWYRQVPGKQRELVAV ITKGGET-----TYEDTVKGRFTISMNSARNSV--YLQMNSLKPADTAVYYC NAKWGYNNSDY------WGQGTQVTVSS
55 34    QVQLVQSGGGLVQAGGSLTLSCSAS GSILRFNS MGWYRQAPGKQRELVAV ITKEGSA-----TYSDSVMGRFTITMNSARNSM--WMQMNSLKPADTAIYYC NAKWGYNNSDY----- WGQGTQVTVSS
58 39    EVQLVESGGGLVQAGGSLRLSCGRS GSFSSLNS MGWFRQAPGKEREFIAS ITIGGGTT----RYADFVKGRFTISRDNGKNTV--YLQMNSLKPEDTAVYYC NADAIVNNRRMQY--- WGQGTQVTVSS
3   210  QVQLVQSGGGLVQAGGSLRLSCAAS GRFFSRYA LGWYRQAPGKQRELVAH ITNGDTT---- DYANSVKGRFTISRDNAKNTG--YLQMNNLKPEDTAVYYC NLGFGGTGGSNSF--- WGQGTQVTVSS(2)
43 255   QVQLVQSGGGLVQPGGSLRLSCAVS GTFSSITA MGWYRQAPGQQRELVAT FTSGGRT---- NYVDSVKGRFTVSRDNARNTVDLYLQMNSLKPEDTAVYYC NVEGLWNNKRERA--- WGQGTQVTVSS
72 340   QVKLVESGGGLVQPGGSLRLSCAVS GTFSSITS MGWYRQAPGKQRELVAT FSGGRTN-----YVDSVKGRFTVSRDNARSTVDL-YLQMNSLKPEDTAVYYC NVEGLWNNRRGRA--- WGQGTQVTVSS
67 325   EVQLVESGGGLVQAGGSLRLSCAAS GSIFSRGNAMAWSRQAPGKQRELVAS ITSDGGA-----YYDDSVKGRFTISADNAKNMV--FLQMNSLKPEDTAVYYC NTLPRRWA-------- WGQGTQVTVSS
4   212  QVQLVQSGGGLVQAGGSLRLSCAAS GNIFSSNAVGWWRRAPGRQQREWVAT ITSGDST---- HYADSVEGRFTISGDNAKNTV--YLQMDSLKPEDTAVYYC TTVPRRGD-------- WGQGTQVTVSS
64  32   EVQLVESGGGLVQAGGSLRLSCAVS GIDFSRYAITWNRQSPGNQRREWVAT LPPADTT-----VYADAVKGRFTISRDNTKNTV--YLQMNSLKPEDTAVYYC ATSPRIHN---------WGQGTQVTVSS
26 230   QVQLVQSGGGSVQAGGSLRLSCAAT RRVYDTKV MGWYRQAPGKQRELVAD FLIINGRVRQP NYAAPVNGRFTISRDSAKDTV--DLQMNSLKPEDTAVYYC SMKLGYPTVSEEY--- WGQGTQVTVSS
34 246   EVQLVESGGGRVQAGGSLRLSCAGS ARLSSIKA MQWSRQAPGKQREWVAT VTPGGST---- IYADSVEGRFTISRDNAKNTV--YLQMDNLKPEDTGMYYC NEMPRIMP-------- WGQGTQVTVSS(3)

32 344   QVKLVESGGGWVQPGGSLRLSCAAS EFTFNTYF MYWARQAPGKGLEWVST ITPGGERT--- VYADSVQGRFTVSRDNAKNTL--YLQMNSLKPEDTALYFC ARGLAAGIANSAAYRY RGQGTQVTVSS
49 273   QVQLVQSGGGLVQPGGSLRLSCTAS GFTFSDAA MVWVRQAPGKGLEWVST IRSDGS-T--- IYAESVKGRFTISRDNAKKTL--YLQMNSLKPEDTAVYYC RRPPMGPDRGDY---- RGQGTQVTVSS
6   224  QVQLVQSGGGLVQPGGSLRLSCAAS GFTFAHYA MTWARQAPGKGLEWVSV INSDGSDT----AYADSVKGRFTISRDNAQSTL--YLQMAGLKPGDTAVYYC SIGRPDWTRE------ RGQGTQVTVSS
2   24   QVQLVQSGGGLVQPGGSLRLSCAAS GFTFANYA MTWYRQAPGKGLEWVSV INSDGSDT--- AYADSVKGRFTISKDNAKNTL--YLQMNSLKPEDTAVYFC GIGRPNWTRE------ RGQGTQVTVSS
57 38    QVQLQESGGGRVQPGGSLRLSCAAS GFTFAHYP MSWVRQAPGKGIEWVSV INSDGSDT----AYADSVKGRFTISRDNAKDTL--YLQMNSLKPEDTAVYYC QIGRPSVTRE------ RGQGTQVTVSS(4)
69 329   EVQLVESGGGLVQPGGSLRLSCAAS GFTFSLYA MNWVRQAPGKGLEWVSV INSDGSDT----AYADSVKGRFTIARDNAKNTL--YLQMNSLKPEDTAVYYC ARGRPGSSSQP----- RGRGTQVTVSS(5)
33 345   EVQLVESGGGLVQPGGSLRLSCAAS GFAFGSYA MTWVRQAPGKGLEWVSV INSDGSDI----AYADSVKGRFTASRDNAKNTL--YLQMNSLKPEDTAVYFC TIGRPTPNWTRE---- RGQGTQVTVSS(2)
```

Figure S3. Capture Nbs to ZVNS1 sequence aligment. The regions corresponding to the frameworks (FR) and the three CDRs are denoted on top. Numbers between brackets indicate the number of clones that have that sequence. Gaps are shown as dashes. The top and bottom sequences derived from VHH and VH genes, respectively. The hallmark residues of VHH are shown in bold and the GLEW motif and the Arg residue that substitus for W in FR4 of VH are also in bold. Sequence clone numbers (plain) and sequence names (bold) are shown on the left.

```
         ----------FR1------------    CDR1      -------FR2------     CDR2       -----------------FR3-------------------    CDR3          ---FR4-----
12 A12   EVQLVESGGGLVQAGGSLRLSCGAS GSFSSINS MGWFRQAPGKERELVAS TTLGGGGA-- KYADSVKDRFTISRDLAKMTV-FLQMSSLKPEDTAVYYC NADGIFNNRRMQY WGQGTQVTVSS
31 C7    QVKLLESGGGLVQAGGSLRLSCGAS GSFSSINS MGWFRQAPGKERELVAS TTLGGGTA-- NYAESVKDRFTISRDLVTMTL-FLQMNSLKPEDTAVYYC NADAIFNNRRMRY WGQGTQVTVSS (2)
20 B8    QVQLVQSGGDLVQPGGSLRLSCAAS GRFFSRYA LGWYRQAPGKQRELVAH ITNGDTT--- DYANSVKGRFTISRDNAQSTG-YLQMNNLKPEDTAVYYC NLGFGGTGGSNSF WGQGTQVTVSS (2)
67 H3    EVQLVESGGGLVQAGGSLRLSCAAS GAIARVNT MAWYRQPSGSQRELVAS WTSTYSEGVT EYADSVKGRFTISRDSAKNT--YLQMKMLRPEDTAVYYC NLEQHGQLRGVY- WGQGTQVTVSS
59 E10   QVKLVESGGGLVQAGGSLRLSCKGS ASVSSNKG MQWSRQAPGKQREWVAT VTPGAST--- IYADSVEGRFTITRDDAQNTV-DLQMDNLQPEDTGIYYC NTMPRTMP----- WGQGTQVTVSS

66 F6    EVQLVESEGGWVQPGGSLRLSCAAS GFTFSLYA MNWVRQAPGKGLEWVSV INSDGSDT-- AYADSVKGRFTIARDNAKNTL-YLQMNSLKPEDTAVYYC ARGRPGSSSQP-- RGRGTQVTVSS (3)
48 E1    QVQLVQSGGGRVQPGGSLRLSCAAS GFTFAHYP MSWVRQAPGKGIEWVSV INSDGSDT-- AYADSVKGRFTISRDNAQDTL-YLQMNSLKPEDTAVYYC QIGRPSVTRE--- RGQGTQVTVSS (2)
7  A7    EVQLVESGGGLVQPGGSLRLSCAAS GFTFAHYA MTWARQAPGKGLEWVSV INSDGSDT-- AYADSVKGRFTISRDNAQSTL-YLQMAGLKPGDTAVYYC SIGRPDWTRE--- RGQGTQVTVSS
42 D6    EVQLVESGGGSVQPGGSLRLSCAAS GFAFSHYA MRWVRQAPGKGLEWVSV INSDGEDT-- WYADSVKGRFTISRDNAKNTL-YLQMNSLKPEDTGVYYC AIGRTHDT----- KGQGTQVTVSS
45 D9    EVQLVESGGGLVQPGGSLRLSCAAS GFAFSHYA MRWLRQAPGKGLDMVSV INSNGDDT-- WYADSVKGRFTISRDNAQNTL-YLQMDMLKPEDTAVYFC AQGRTHDT----- KGQGTQVTVSS (2)
```

Figure S4. Detection Nbs to ZVNS1 sequence aligment. The regions corresponding to the frameworks (FR) and the three CDRs are denoted on top. Numbers between brackets indicate the number of clones that have that sequence. Gaps are shown as dashes. The top and bottom sequences derived from VHH and VH genes, respectively. The hallmark residues of VHH are shown in bold and the GLEW motif and the Arg residue that substitus for W in FR4 of VH are also in bold. Sequence clone numbers (plain) and sequence names (bold) are shown on the left.

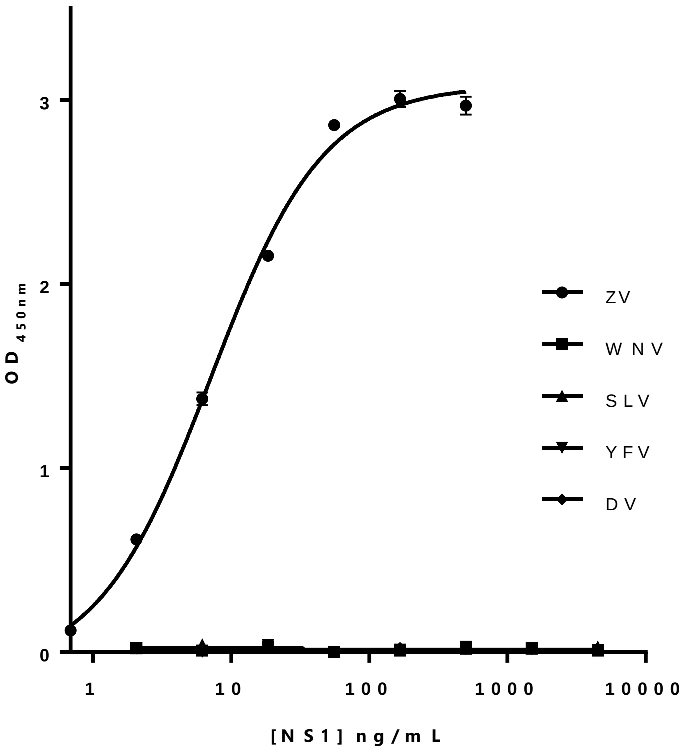

Figure S5. Analysis of cross-reactivity of the 32/D6 nanobody pair with non-Zika NS1. Each NS1 was individually tested in the 0.3-4500 ng/mL range. The results are the average values of triplicate measurements and the error bars represent the standard deviation.
